# Pediatric methylation class HGNET-*MN1*: unresolved issues with terminology and grading

**DOI:** 10.1186/s40478-019-0834-z

**Published:** 2019-11-10

**Authors:** Arnault Tauziède-Espariat, Mélanie Pagès, Alexandre Roux, Aurore Siegfried, Emmanuelle Uro-Coste, Yvan Nicaise, Annick Sevely, Marion Gambart, Sergio Boetto, Martin Dupuy, Pomone Richard, Romain Perbet, Matthieu Vinchon, Sabine Caron, Felipe Andreiuolo, Albane Gareton, Emmanuèle Lechapt, Fabrice Chrétien, Stéphanie Puget, Jacques Grill, Nathalie Boddaert, Pascale Varlet, on behalf of the RENOCLIP-LOC

**Affiliations:** 1 Department of Neuropathology, GHU Paris Psychiatrie Neurosciences, Sainte-Anne Hospital, 1, rue Cabanis, 75014 Paris, France; 2 Paris University France, 75006 Paris, France; 3 Department of Neurosurgery, GHU Paris Psychiatrie Neurosciences, Sainte-Anne Hospital, 75014 Paris, France; 4 Department of Pathology, Toulouse University Hospital, 31300 Toulouse, France; 5 Department of Radiology, Purpan University Hospital, 31300 Toulouse, France; 6 Department of Pediatric Oncology, Toulouse University Hospital, 31300 Toulouse, France; 7 Department of Neurosurgery, Toulouse University Hospital, 31300 Toulouse, France; 8 Department of Neurosurgery, Clinique de l’Union, 31240 Saint-Jean, France; 9 Laboratory of Pathology, Les Feuillants, 31023 Toulouse, France; 10 Institute of Pathology, Centre de Biologie Pathologie, Lille University Hospital, 59000 Lille, France; 11 Neurology, Breast Cancer Department, Oscar Lambret Center, 59000 Lille, France; 12 Department of Radiology, Centre de Biologie Pathologie, Lille University Hospital, 59000 Lille, France; 13 Department of Pediatric Neurosurgery, Necker University Hospital, 75015 Paris, France; 14 CNRS, Gustave Roussy, University Paris-Sud, Université Paris-Saclay, UMR8203 “Vectorologie et Thérapeutiques Anticancéreuses”, 94805 Villejuif, France; 15 Department of Pediatric Oncology, Gustave Roussy, University Paris-Sud, Université Paris-Saclay, 94805 Villejuif, France; 16 Department of Radiology, Necker University Hospital, 75015 Paris, France

**Keywords:** MN1, HGNET, Astroblastoma, Meta-analysis

High-grade neuroepithelial tumor with *MN1* alteration (HGNET-*MN1*) is a rare, recently described central nervous system (CNS) entity with a distinct methylation profile, which was formerly part of CNS-primary neuroepithelial tumors (PNET). HGNET-*MN1* affects children (77% of reported cases [1–5]) and are characterized by a recurrent fusion implicating the *MN1* (meningioma 1) gene [1]. Limited histopathological and clinical data are available on HGNET-*MN1* (44 cases proven by DNA methylation analysis) and a number of outstanding issues still exist [1–5].

Firstly, the histopathological diagnostic criteria are not well established. In Sturm’s study, they were initially classified as astroblastomas (16/41, as 4 of our cases) but also as PNETs (12/41), ependymomas (9/41, as 6 of our cases), meningioma (1/41), glioblastoma (1/41), embryonal tumor with multilayered rosettes (1/41), and unclassified tumor (1/41) [1]. These data raise the question of an overlap between a histopathological entity (astroblastoma, considered as a glioma in the current World Health Organization –WHO- classification) and a molecular entity (HGNET-*MN1)*. Secondly, the prognosis of HGNET-*MN1* remains unclear. The term “HG” was employed because they were initially described within a cohort of malignant tumors [1]. To explore these issues, we screened our local and the French neuropathological network database (*n* = 10, five of them were briefly mentioned in [6]) and the literature (*n* = 34), for pediatric DNA-methylation proven HGNET-*MN1* [1–5]. Here, we describe the molecular, histopathological, clinical and imaging characteristics of pediatric HGNET-*MN1*.

Including our cases, HGNET-*MN1* predominantly concern girls (90.9%, 40/44 cases) and are mainly supra-tentorial (95.3%, 41/43 cases) [1, 3–5]. Radiologically, all our tumors were well-demarcated from adjacent parenchyma with a multi-nodular aspect. Perilesional edema was not constantly observed. None was calcified and one case was hemorrhagic. They consisted of very large lesions (size ranging from 4.5 to 11.0 cm, mean 7.1 cm) with prominent solid portions and multiple necrotic areas but a unique cystic component was not the main tumor feature (Additional file 1: Figure S1 A-D). Thus, the radiological analysis showed that HGNET-*MN1* differed from ependymoma, with *RELA*-fusion, the main pediatric differential diagnosis [6].

In the literature, HGNET-*MN1* has been identified in a portion of astroblastomas (39% in adults compared to 78% in children) and did not constantly harbour astroblastic features (one case of our series and 8.5% of reported pediatric cases) [1–5]. In our series, the only constant feature was tumoral fibrous or sclerous stroma (Additional file 1 Figure S1 E-F). They were often macro- and microcystic (*n* = 7) (Additional file 1 Figure S1 G-H). They showed a large variety of architectural patterns (Additional file 2 Figure S2 A-E). Cellular atypia was mild except in two cases (Additional file 2: Figure S2 F). True ependymal rosettes, lymphocytic infiltrates, eosinophilic granular bodies, and Rosenthal fibers were absent. Mitotic count ranged from 1 to 48 for 10 high-power fields (HPF) and the MIB1 ranged from 2 to 40% (Fig. 1). Microvascular proliferation and necrosis were observed in 3 and 8 cases, respectively (Fig. 1). The immunohistochemical profile (summarized in Additional file 5 Table S1 and Additional file 3: Figure S3) is also heterogeneous. The literature review and our cases show that 72% of tumors express glial markers [1–5] and 58% express neuronal markers [1, 5]. Our analysis revealed that 50% of cases express CK18, and, surprisingly for a primary brain tumor, CD56 was focally or not expressed in 5/10 tumors. Further fundamental studies and ultrastructural analyses are required to elucidate the cell of origin of HGNET-*MN1*. Thus, HGNET-*MN1* present variable morphological and immunophenotypical features.

**Fig. 1:**
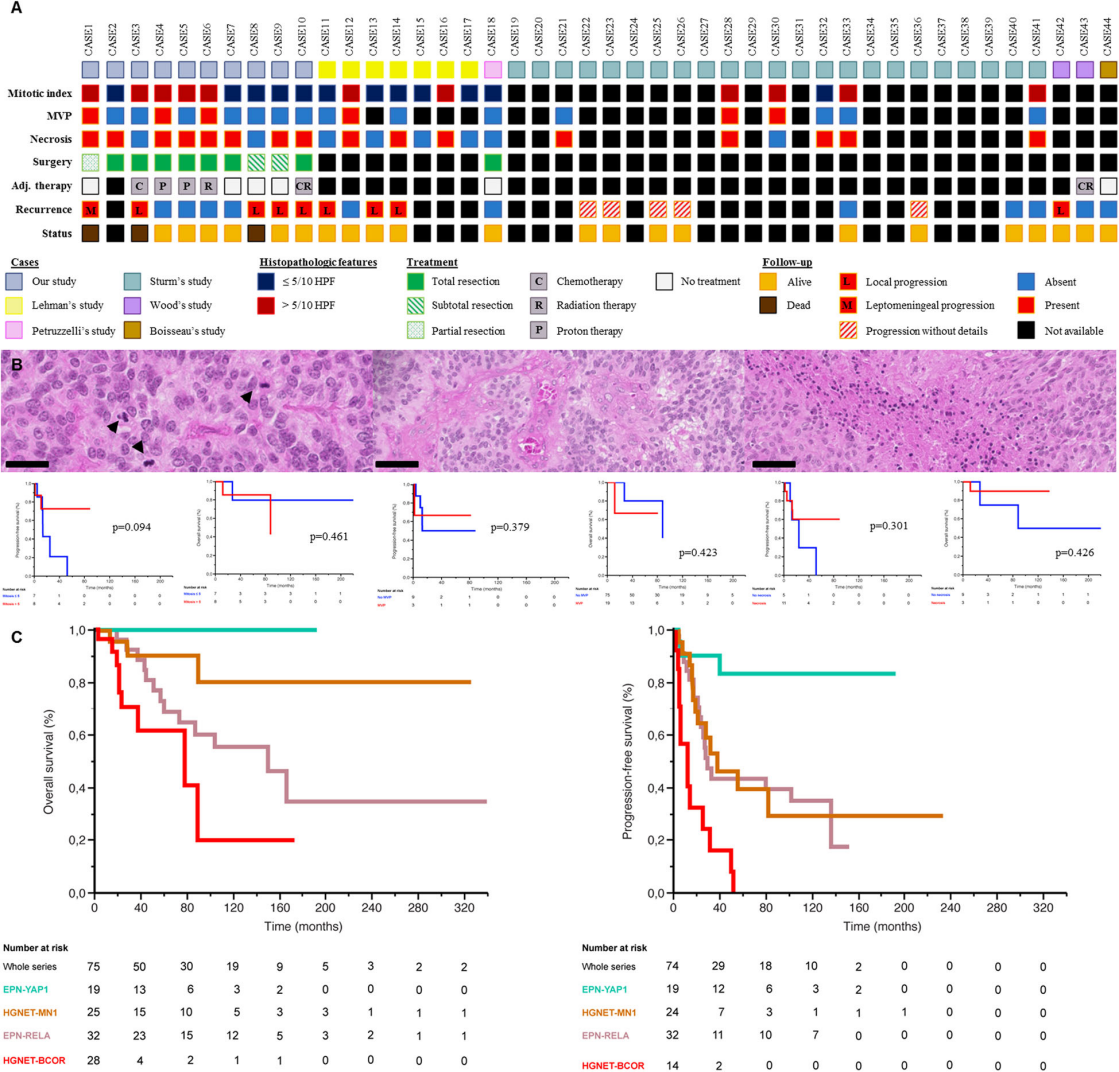
Results of the meta-analysis and prognostic data of HGNET-*MN1.* (**a**) Integrative annotation of histopathologic features (mitotic index, microvascular proliferation -MVP- and necrosis), treatment (surgery and adjuvant therapy -adj. Therapy-) and follow-up of our cases and cases of the literature included in the meta-analysis (**b**) There is no significant difference in terms of PFS and OS between HGNET-*MN1* with or without elevated mitotic index (*p* = 0.094 and 0.461 respectively), microvascular proliferation (*p* = 0.379 and 0.423, respectively) and necrosis (*p* = 0.301 and 0.426, respectively).(**c**) Results of the meta-analysis including 32 ependymomas, *RELA*-fusion (EPN-RELA, *n* = 32), HGENT-*MN1* (*n* = 24), 19 ependymomas, *YAP1-*fusion (EPN-YAP1, *n* = 19), and HGNET-*BCOR* (*n* = 28). Kaplan–Meier estimates of overall survival (OS) and progression free survival (PFS) according to DNA-methylation subgroups: There is a significant difference in terms of PFS (*p* < 0.001) and OS (p < 0.001) between EPN-RELA, EPN-YAP1, HGNET-*BCOR* and HGNET-*MN1*

Our meta-analysis (summarized in Fig. 1**,** with statistical methodology detailed in **Supplementary Data)**, revealed that during follow-up (mean 81.4 months, range 2.6–324.0), 14/25 patients (56.0%) experienced tumor progression (8 local, 1 leptomeningeal, and 5 without details) and 3 patients (12.0%) died (Fig. 1**)**. The mean/median progression-free survival (PFS) were 43.9/34 months. The mean overall survival (OS) was 81.6 months and the median OS was not reached. Children with HGNET-*MN1* present a favourable outcome with 88.0% survival (median follow-up of 65.5 months), although 48.0% presented with tumor progression. Three patients (cases 7, 18 and 44) treated only by surgery were alive without tumor progression: one with a long follow-up (229 months) and two with only a short follow-up period (20 and 21 months). Total resection was significantly associated with better OS (*p* = 0.032) and PFS (*p* = 0.029). In univariate analysis, we found significant differences in terms of OS (*p* < 0.001) and PFS (p < 0.001) between HGNET-*MN1* and ependymomas with *RELA-*fusion, ependymomas with *YAP1*-fusion and HGNET-*BCOR* from previous studies [1, 6–9]. Our study has several limitations inherent to its retrospective observational design, but the prognosis does not seem to be as dismal as the term “high-grade” could suggest. No grading criteria have been established for HGNET-*MN1*. However, the WHO classification mentions histopathologic criteria for malignant astroblastoma [10]: mitotic index > 5/10 HPF, microvascular proliferation and palisading necrosis. In univariate analysis, these criteria were not significantly, independently or in combination, associated with worse OS (*p* = 0.461, 0.423, 0.426 respectively and in combination p = 0.461) and PFS (*p* = 0.094, 0.379, 0.301 respectively and in combination *p* = 0.426) (Fig. 1, Additional file 4: Figure S4). Moreover, three patients (cases 4, 6 and 12) presenting tumors with these three criteria did not develop progression and are alive (median follow-up of 68.0 months) [5].

In summary, this is the largest series of pediatric HGNET-*MN1* with clinical, radiological and histopathological characterization. They present a homogeneous MRI aspect, but a heterogeneous morphology, with inconstant astroblastic pseudorosettes. *MN1* fusion must be confirmed to rule out the major differential diagnoses (pleiomorphic xanthoastrocytomas [2–4] and ependymomas [3]). Nevertheless when confronted to our metaanalysis results, naming these tumors has proven difficult. Indeed, both “HGNET” and “astroblastoma” do not completely correlate with grading and morphology. “HGNET” is not a proper designation because the prognosis seems favourable in a subset of cases and further studies are needed to comfort this tendency. “Astroblastoma” is also not quite appropriate since astroblastic pseudorosettes are inconstant and not specific, and a frequent glioneuronal immunophenotype is observed. Our study showed that the two constant histopathological and molecular features of these tumors are the fibrous stroma and the *MN1* fusion. Consequently, we would like to suggest the terms “NET, *MN1* fusion-positive” or “Fibrous neuroepithelial tumors (FNET), *MN1* fusion-positive”.

## Supplementary information

**Additional file 1: Figure S1.** Correlation of radiological and morphological features in HGNET-*MN1.* (**A**) Axial T1 weighted image; (**B**) Axial T1 post contrast weighted image; (**C**) Axial T2 weighted image; (**D**) Coronal T1 post contrast weighted image: they showed a large lesion with multinodular appearance and very important edema. It is a well-demarcated non-intraventricular multinodular tumor with a central solid portion (yellow star) surrounded by multiple cystic components (red asterisks). (**E**) Multilobular tumor with fibrous central scar delimiting tumor nodules (HPS, 60x). (**F**) Fibrous scar (yellow star) (HPS, 130x). (**G**) Macro- and microcystic components (red asterisks) (HPS, 100x). (**H**) Well-delimitation of the tumor from the brain parenchyma (arrows) (HPS, 200x).

**Additional file 2: Figure S2.** Variable histopathological patterns of HGNET-*MN1.* (**A**) Astroblastic pseudorosettes with variable hyalinization of vessels (HPS, 300x). (**B**) Ependymoma-like pattern with pseudorosettes (HPS, 400x). (**C**) Trabecular pattern of the tumor outside of the pseudorosettes (HPS, 400x). (**D**) Fibrous sclerosis with cordonal structures (HPS, 400x). (**E**) Myxoid changes with chordoid appearance (HPS, 400x). (**F**) Tumor cells with nuclear inclusions, atypias and giant cells (HPS, 400x).

**Additional file 3: Figure S3.** Variable immunohistochemical findings of HGNET-*MN1.* (**A**) Diffuse expression of GFAP by glial cells of the pseudorosettes (400x, and insert 400x). (**B**) Focal expression of GFAP (400x). (**C**) Diffuse expression of Olig2 in a part of the tumor (400x). (**D**) Very focal immunoreactivity for Olig2 (400x). (**E**) Expression of CD56 (400x). (**F**) Expression of synaptophysin in a part of tumor cells (400x). (**G**) NeuN immunopositivity (400x). (**H**) Extra-vascular cellular staining with CD34 in one case (400x). (**I**) Diffuse expression of cytokeratin 18 (400x).

**Additional file 4: Figure S4.** Analysis of correlation between malignant criteria defined in astroblastoma applied to HGNET-*MN1* with overall survival and progression-free survival. (**A**) There is no significant difference in terms of PFS between HGNET-*MN1* with or without the combination of the three features of anaplasia (*p* = 0.426). (**B**) There is no significant difference in terms of OS between HGNET-*MN1* with or without the combination of the three features of anaplasia (*p* = 0.461).

**Additional file 5: Table S1.** Immunoprofile of HGNET-*MN1* of our series
