# Supplementary figures and images for: Pediatric methylation class HGNET-MN1: unresolved issues with terminology and grading
Source: Acta Neuropathol Commun. 2019 Nov 10;7:176. doi: 10.1186/s40478-019-0834-z (PMC6842469; doi:10.1186/s40478-019-0834-z)

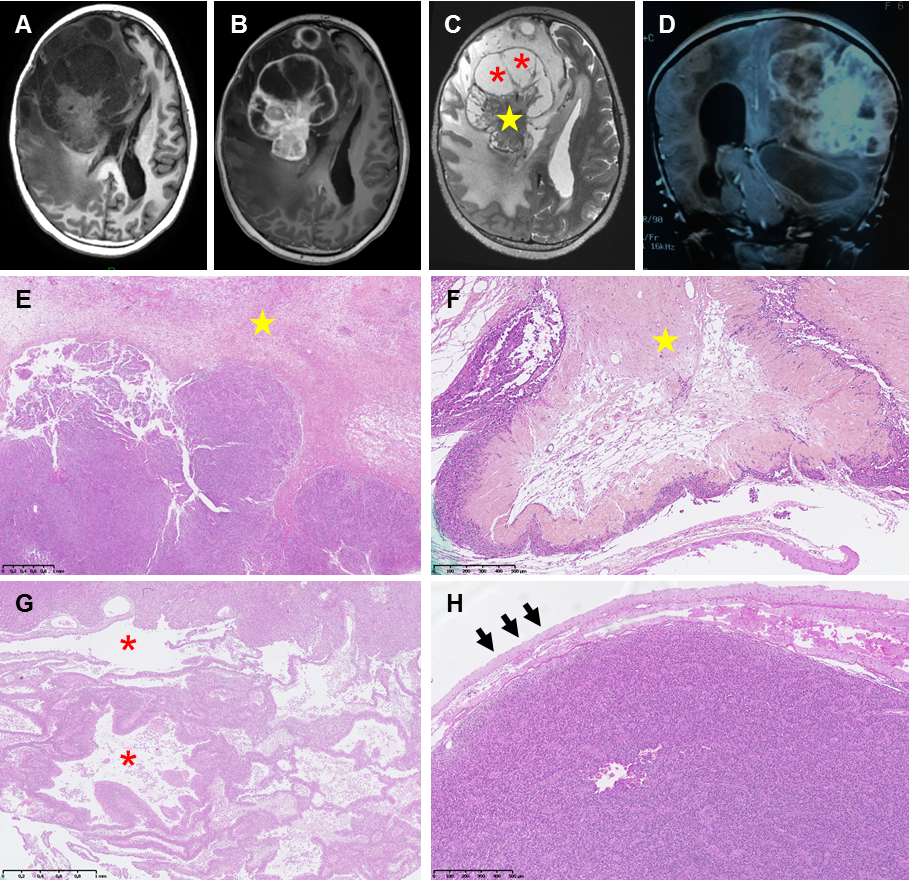

Supplement: Supplementary file 1 — Additional file 1: Figure S1. Correlation of radiological and morphological features in HGNET-MN1. (A) Axial T1 weighted image; (B) Axial T1 post contrast weighted image; (C) Axial T2 weighted image; (D) Coronal T1 post contrast weighted image: they showed a large lesion with multinodular appearance and very important edema. It is a well-demarcated non-intraventricular multinodular tumor with a central solid portion (yellow star) surrounded by multiple cystic components (red asterisks). (E) Multilobular tumor with fibrous central scar delimiting tumor nodules (HPS, 60x). (F) Fibrous scar (yellow star) (HPS, 130x). (G) Macro- and microcystic components (red asterisks) (HPS, 100x). (H) Well-delimitation of the tumor from the brain parenchyma (arrows) (HPS, 200x). [file 40478_2019_834_MOESM1_ESM.tif]

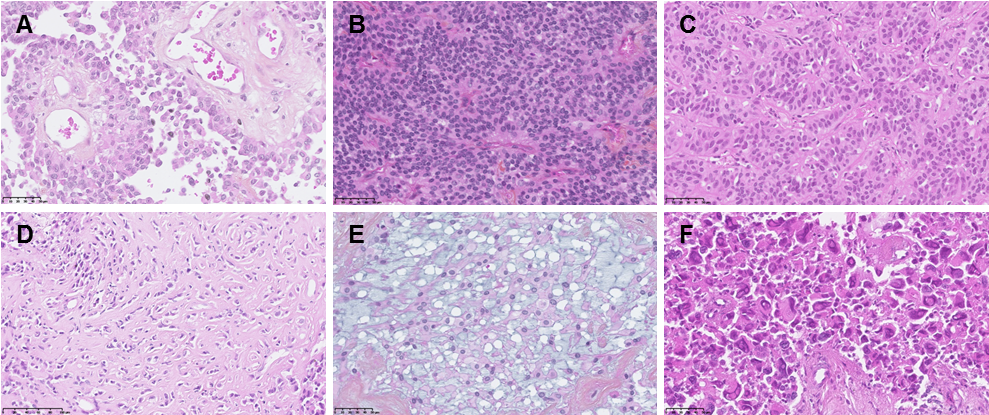

Supplement: Supplementary file 2 — Additional file 2: Figure S2. Variable histopathological patterns of HGNET-MN1. (A) Astroblastic pseudorosettes with variable hyalinization of vessels (HPS, 300x). (B) Ependymoma-like pattern with pseudorosettes (HPS, 400x). (C) Trabecular pattern of the tumor outside of the pseudorosettes (HPS, 400x). (D) Fibrous sclerosis with cordonal structures (HPS, 400x). (E) Myxoid changes with chordoid appearance (HPS, 400x). (F) Tumor cells with nuclear inclusions, atypias and giant cells (HPS, 400x). [file 40478_2019_834_MOESM2_ESM.tif]

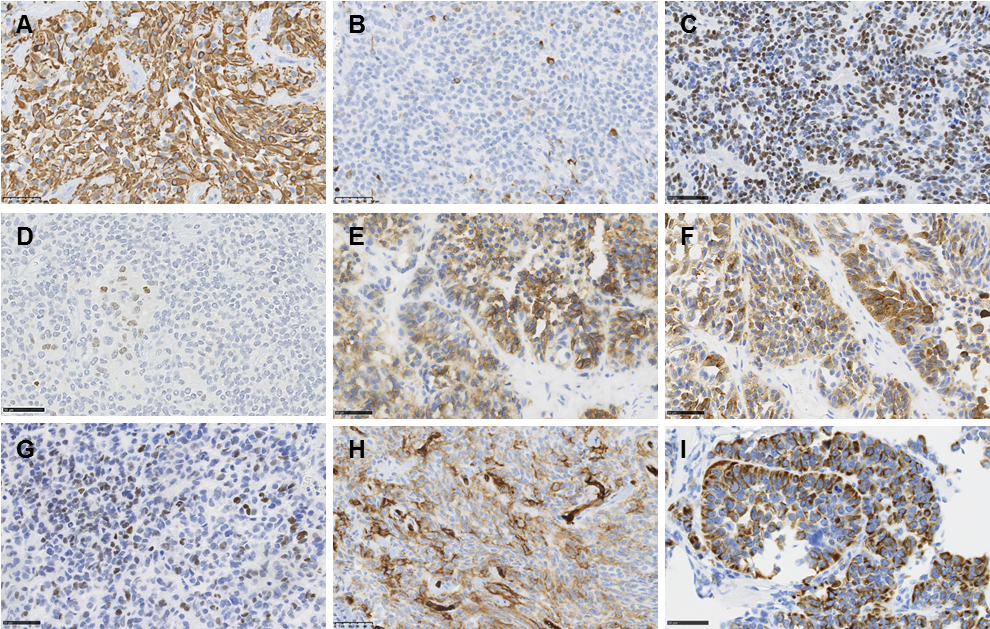

Supplement: Supplementary file 3 — Additional file 3: Figure S3. Variable immunohistochemical findings of HGNET-MN1. (A) Diffuse expression of GFAP by glial cells of the pseudorosettes (400x, and insert 400x). (B) Focal expression of GFAP (400x). (C) Diffuse expression of Olig2 in a part of the tumor (400x). (D) Very focal immunoreactivity for Olig2 (400x). (E) Expression of CD56 (400x). (F) Expression of synaptophysin in a part of tumor cells (400x). (G) NeuN immunopositivity (400x). (H) Extra-vascular cellular staining with CD34 in one case (400x). (I) Diffuse expression of cytokeratin 18 (400x). [file 40478_2019_834_MOESM3_ESM.tif]

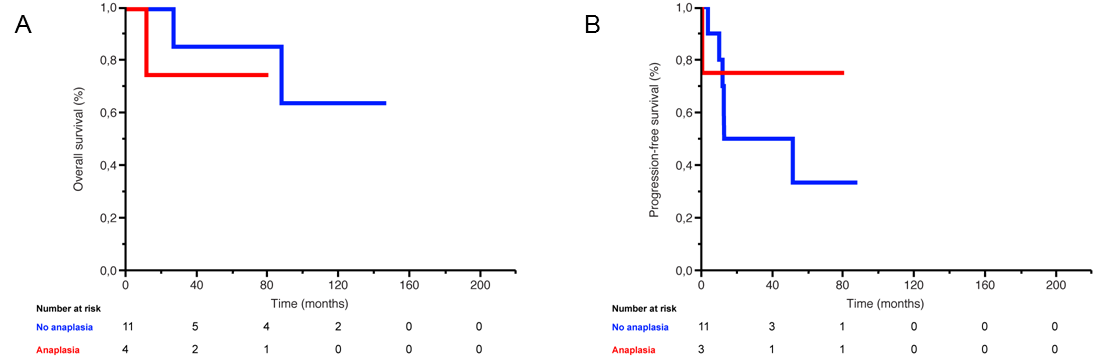

Supplement: Supplementary file 4 — Additional file 4: Figure S4. Analysis of correlation between malignant criteria defined in astroblastoma applied to HGNET-MN1 with overall survival and progression-free survival. (A) There is no significant difference in terms of PFS between HGNET-MN1 with or without the combination of the three features of anaplasia (p = 0.426). (B) There is no significant difference in terms of OS between HGNET-MN1 with or without the combination of the three features of anaplasia (p = 0.461). [file 40478_2019_834_MOESM4_ESM.tif]
