# Supplementary material for: Pediatric methylation class HGNET-MN1: unresolved issues with terminology and grading
Source: Acta Neuropathol Commun. 2019 Nov 10;7:176. doi: 10.1186/s40478-019-0834-z (PMC6842469; doi:10.1186/s40478-019-0834-z)
Supplement: Supplementary file 5 — Additional file 5: Table S1. Immunoprofile of HGNET-MN1 of our series [file 40478_2019_834_MOESM5_ESM.doc]

**Table 1. Immunoprofile of HGNET-*MN1* of our series**

| **Case** | **CD56** | **GFAP** | **Olig2** | **EMA** | **NFκB** | **CKAE1/AE3** | **NeuN** | **Synaptophysin** | **Chromogranin A** | **CK18** | **Actin** | **CD34** | **BRAFV600E** | **p53** |
| --- | --- | --- | --- | --- | --- | --- | --- | --- | --- | --- | --- | --- | --- | --- |
| **1** | **-** | **++** | **++** | **++c,d** | **+++c** | **-** | **++** | **++** | **-** | **-** | **-** | **-** | **-** | **-** |
| **2** | **+++** | **++** | **++** | **++c,d** | **+++c** | **-** | **-** | **-** | **-** | **-** | **-** | **-** | **-** | **-** |
| **3** | **+++** | **++** | **-** | **+c** | **+++c** | **-** | **-** | **-** | **-** | **++** | **-** | **-** | **-** | **-** |
| **4** | **+** | **++** | **+** | **++c,d,m** | **+++c** | **-** | **-** | **-** | **-** | **++** | **-** | **-** | **-** | **-** |
| **5** | **-** | **+++** | **-** | **++c,d** | **+++c** | **-** | **-** | **-** | **-** | **-** | **-** | **-** | **-** | **-** |
| **6** | **+** | **++** | **+** | **++c, d, m** | **+++c** | **-** | **+** | **++** | **-** | **-** | **-** | **+++** | **-** | **+++** |
| **7** | **+** | **+** | **+** | **++c, d, m** | **+++c** | **-** | **-** | **-** | **-** | **-** | **-** | **-** | **-** | **-** |
| **8** | **++** | **-** | **+** | **++c, d, m** | **+++c** | **-** | **-** | **-** | **-** | **++** | **-** | **-** | **-** | **-** |
| **9** | **++** | **++** | **+** | **++c, m** | **+++c** | **-** | **-** | **++** | **-** | **++** | **-** | **-** | **-** | **++** |
| **10** | **+++** | **-** | **-** | **+c** | **+++c** | **-** | **-** | **-** | **-** | **+** | **-** | **-** | **-** | **-** |

**+: focal staining; ++: patchy staining; +++: diffuse staining; c: cytoplasmic; d: dot staining; m: membranous staining.**
